# Response to “Old Black Water”

**Published:** 2005-12-15

**Authors:** Jane A Zanca

**Affiliations:** Office of Enterprise Communications, Centers for Disease Control and Prevention, Atlanta, Ga

## To the Editor:

Thanks for the wonderful item on the 1927 Mississippi River flood (1). I grew up in New Orleans and on the Gulf Coast of Mississippi, hearing of storms and wading through floods all of my life, but no one had ever talked about the 1927 events, and this critical southern event was not taught in any history courses at my schools. Reading John Barry's *Rising Tide: The Great Mississippi Flood of 1927 and How It Changed America* (2) was an epiphany and a joyous discovery. I had always wondered what it was like at the bottom of the river; he could answer that question.

Barry also describes deliberate destruction of the levee along St. Bernard Parish to spare New Orleans from the rising tide in the 1927 flood. I had heard rumors of the destruction — but no mention of the flood in 1927 — for years but had never seen documentation to support it until Barry's book. The rumors reemerged in Hurricane Betsy, which drove my family out of their home in Arabi in the middle of the night with flooding from the Lake Pontchartrain end of the Industrial Canal. Many in the flooded communities below the canal firmly believed the levee had been bombed — once again to spare New Orleans at the expense of the 9th Ward, Arabi, Chalmette, and other areas. An Arabi neighbor told me that in the decades after Hurricane Betsy, any time a storm was threatening New Orleans, vigilantes from below the Industrial Canal patrolled the levees. I can't testify to that — but I wouldn't be surprised.

**Jane A. Zanca** Technical Writer and Editor, Office of Enterprise Communications, Centers for Disease Control and Prevention, Atlanta, Ga
